# The Application of Surface Luminance Distribution Measurements to the Evaluation of Neoplastic Lesions of the Prostate Gland

**DOI:** 10.3390/cancers17040639

**Published:** 2025-02-14

**Authors:** Krzysztof Tereszkiewicz, David Aebisher, Henryk Wachta, Łukasz Kulig, Michał Osuchowski, Ewa Kaznowska, Wojciech Domka, Mateusz Polar, Angelika Myśliwiec, Klaudia Dynarowicz, Dorota Bartusik-Aebisher

**Affiliations:** 1 Department of Computer Engineering in Management, Faculty of Management, Rzeszow University of Technology, Powstańców Warszawy 10, 35-959 Rzeszów, Poland; kteresz@prz.edu.pl (K.T.); m.polar@prz.edu.pl (M.P.); 2 Department of Photomedicine and Physical Chemistry, Collegium Medicum, University of Rzeszow, 35-959 Rzeszów, Poland; daebisher@ur.edu.pl; 3 Department of Power Electronics and Power Engineering, Faculty of Electrical and Computer Engineering, Rzeszow University of Technology, Pola 2, 35-959 Rzeszow, Poland; hwachta@prz.edu.pl; 4 Department of Pathomorphology, Collegium Medicum, University of Rzeszow, 35-959 Rzeszów, Poland; mosuchowski@ur.edu.pl (M.O.); ekaznowska@ur.edu.pl (E.K.); 5 Department of Otolaryngology, Collegium Medicum, University of Rzeszów, 35-959 Rzeszów, Poland; wdomka@ur.edu.pl; 6 Department of Biochemistry and General Chemistry, Collegium Medicum, University of Rzeszów, 35-959 Rzeszów, Poland; amysliwiec@ur.edu.pl (A.M.); kdynarowicz@ur.edu.pl (K.D.); dbartusikaebisher@ur.edu.pl (D.B.-A.)

**Keywords:** luminance distribution measurements, prostate cancer, surface image analysis, medical diagnostics, measurements using computer applications

## Abstract

**Objective:** The aim of the retrospective study undertaken was to assess the feasibility of using an integrated bench for the measurement and analysis of surface luminance distribution to identify neoplastic lesions in samples collected from prostate glands. **Methods:** The material for the study consisted of tissue sections taken from prostate glands removed during radical prostatectomy. Tissues were collected from 30 patients aged 55–80 years with localised prostate cancer (well-differentiated prostate adenocarcinoma) with a TNM pathological stage of pT2c. Two samples were taken from each patient, one containing cancerous tissue and one with benign hyperplasia. 27 out of 30 samples with cancerous tissue and 21 out of 30 samples with benign hyperplasia qualified for the experiment. Unfixed and unstained samples were used in the experiment. A measurement of the surface luminance distribution was recorded on the samples and analysed. The results were statistically analysed to determine the compliance of the results obtained by the surface luminance distribution measurement and by microscopic histopathological analysis. The strength of the relationship between the methods was determined by the sensitivity and specificity index using a 95% confidence interval. **Results:** The study showed significant differences between the arithmetic means of the maximum luminance recorded on the surface of samples containing cancerous tissue (1188.4 cd/m^2^, SD = 19.02) and without neoplastic lesions (1091 cd/m^2^, SD = 27.3). Statistical analysis confirmed that the difference between the means was statistically significant. The highest sensitivity and specificity values were obtained for a luminance threshold of Lmax = 1100 cd/m^2^. The reliability of the test for the designated threshold was 87.50%. It is worth noting the very high probability of a positive result obtained with the luminance measurement being as high as 95.65% compatible with a positive result obtained with the reference histopathological examination. **Conclusions**: The study showed significant differences in the maximum luminance values for samples that contained cancerous tissue and samples without neoplastic lesions, which allows for their differentiation. It has been proven that the method tested has a high reliability index of 87.50% and a 95.65% probability of compatibility with the results of the reference histopathological examination.

## Simple Summary

Prostate cancer is one of the most commonly diagnosed malignancies in men. An important role in the successful fight against prostate cancer is played by modern diagnostic methods. The aim of the study undertaken was to assess the feasibility of using an integrated bench for the measurement and analysis of surface luminance distribution to identify neoplastic lesions in samples taken from prostate glands. Tissue sections removed during radical prostatectomy procedures were used in the study. The results were compared with microscopic histopathological examination as a reference test. The tests carried out showed that the surface luminance distribution analysis method allows the identification of neoplastic structural tissue changes in prostate samples. The method has a high reliability index of 87.50% and a 95.65% probability of compatibility with the results of the reference histopathological examination. The results indicate that the method can be a tool to help diagnose prostate cancer.

## 1. Introduction

Prostate cancer is one of the most commonly diagnosed malignancies in men. The disease is global in scope [1,2,3]. According to the Union for International Cancer Control (UICC), there were more than 1.414 million prostate cancer diagnoses worldwide in 2020, and the number of new cases is on an upward trend. Numerous publications show that the number of prostate cancer diagnoses is increasing particularly rapidly in Western countries. In the United States, more than 200,000 cases of prostate cancer are diagnosed each year [3]. In Poland, according to data from the National Cancer Registry [4], an incidence of 17,638 cases was reported in 2019 and an increase of 1224 cases was observed compared to 2018. The prostate cancer mortality rate in Poland was 5618, with a total number of cancer deaths of 54370. 

Changing demographics, ethnicity, genetic predisposition, lifestyle, physical activity, preferred diet and, in particular, the progressive ageing of the population have been identified as the main reasons for the high incidence and increasing trend of prostate cancer occurrence [2,5]. In the majority of cases, the disease is diagnosed above 65 years of age, when patients present to their doctors because of the presenting symptoms accompanying prostatic hyperplasia [2,6,7]. The implementation of numerous education, prevention and screening programmes has contributed to the detection of PCa and the identification of risk factors [8,9]. At the same time, it should be noted that not all factors responsible for the increase in prostate cancer cases have been fully defined [10]. Multidirectional research is still needed to identify risk factors for the disease and to implement diagnostic methods to detect the disease in its early stages. It should also be emphasised that prostate cancer diagnosed early is fully curable. And for the treatment of advanced stages of the disease, the available therapies significantly extend the life expectancy of patients [11].

An important role in the successful fight against prostate cancer is played by modern diagnostic methods used in the prevention, treatment and systematic follow-up of the patient after therapy. 

Numerous methods are used in the diagnosis of prostate cancer (PCa) [9,12]. The diagnosis of prostate cancer is primarily based on a laboratory test of the level of prostate-specific antigen, i.e., PSA in blood serum [13]. The biomarker allows for the quick and easy initial diagnosis of prostate cancer. It is believed [9] that screening and improvements in the treatment of PCa may contribute to a downward trend in overall mortality, which is unrelated to the number of diagnosed cases. Organised screening has been shown to improve survival rates in men aged 50–69 years by 20% [8,14]. The literature [11] shows that new factors are being explored as potential biomarkers to classify and assess the severity of the disease.

A prostate biopsy is recommended when the PSA level is ≥3 ng/ml or when lesions are suspected on rectal palpation (DRE) [8]. Histopathological examinations of the collected tissue allow for the detection of local neoplastic lesions, a microscopic evaluation of the tissue structure of the cancer and the determination of the aggressiveness of the cancer based on the Gleason scale [15]. Biopsies are an invasive procedure with a high risk of complications including rectal bleeding, haematuria and sepsis [16]. For these reasons, modern diagnostics is moving towards non-invasive methods using Computer Vision Systems—CVSs. Images obtained from digital cameras and scanners provide information on external features. On the other hand, ultrasound, NMR, CT and DXA methods have made it possible to obtain information on the internal structures of organs and tissues [12,17,18]. A particularly useful and commonly used CVS technique for the diagnosis of prostate cancer is transrectal ultrasonography (TRUS) [19]. It is currently used mainly for core biopsies under TRUS guidance [20]. The most accurate imaging test to assess neoplastic lesions in the prostate gland is MRI [9,15,21]. The current 3-Tesla MRI generates high-resolution images that are able to identify small cancer foci that are not visible on TRUS [16,22]. Multi-parametric MRI (MP-MRI) shows how large the cancer is, helps determine how aggressive the cancer is [23] and significantly reduces the need for biopsy [16,22]. The main surgical treatment for prostate cancer is radical prostatectomy [24], which is widely regarded as the ‘gold standard’ for the treatment of prostate cancer [25].

Surface luminance distribution is among the parameters used and interpreted by computer image analysis systems. The SI measure of luminance according to PN-90/E-01005 is candela/m^2^. A luminance meter is used to measure luminance [26]. Previous results from our own research [27] allow us to conclude that the measurement of surface luminance distribution can be used to assess the structure and morphological components of animal tissues. 

The above has inspired research into the possibility of using surface luminance distribution measurements to identify neoplastic lesions, and it can be assumed that the use of surface luminance distribution to assess neoplastic lesions in specimens subject to histopathological analysis could be a useful tool to aid the work of oncology pathologists, with the option of incorporating artificial intelligence [28].

The aim of the study undertaken was to assess the feasibility of using an integrated bench for the measurement and analysis of surface luminance distribution to identify neoplastic lesions in samples taken from prostate glands. The method uses the phenomenon of the differentiated luminance of ex vivo tissue structures showing no lesions and those showing neoplastic lesions. The results were compared with microscopic histopathological examination as a reference test. It is assumed that positive results from verification of the method’s efficacy allow its use in histopathological studies.

The authors’ solution in the form of the measuring test bench and the method of performing the measurement have been applied for patent protection at the Patent Office of the Republic of Poland (patent application no: P.444909 dated 16 May 2023, patent application no: P.445483 of 5 July 2023).

## 2. Materials and Methods

Thirty patients aged 55–80 years who were diagnosed with prostate cancer on the basis of a core biopsy and were qualified for surgical treatment by the Department of Urology at the Clinical Hospital No. 1 in Rzeszów (Podkarpackie Province, Poland) were selected for the experiment. All patients signed a written informed consent form. The study received a positive opinion from the Bioethics Committee of the Rzeszów Regional Medical Chamber (*Resolution No. 22/2023/B of the Bioethics Committee of the Regional Medical Chamber of 13 March 2023*). Tissues were taken from patients with localised prostate cancer (well-differentiated prostate adenocarcinoma). Two samples were collected after radical prostatectomy from each patient, one containing cancerous tissue and one with benign hyperplasia. 27 out of 30 samples with cancerous tissue and 21 out of 30 samples with hyperplasia qualified for the experiment. Two tissue specimens were taken from one of the lobes, one from the peripheral part (where cancer usually develops) and one from the central part (as so-called healthy tissue). Fragments with an average size of 8 × 4 × 4 mm were taken from the peripheral part of the organ (due to the much higher incidence of cancers in this area), and the second fragment was taken from the central part of the organ, with a high probability of benign nodular hyperplasia, with average dimensions of 5 × 3 × 2 mm. Immediately after collection, the specimens were referred to the histopathology laboratory. Unfixed and unstained samples were used in the experiment. The grade of the neoplastic lesions was determined by microscopic examination using the Gleason scale. Specimens with scores of 3 + 3 (grade 1) and 3 + 4 (grade 2) were selected for the experiments according to the TNM pathomorphological classification—pT2c. 

**Figure 1:**
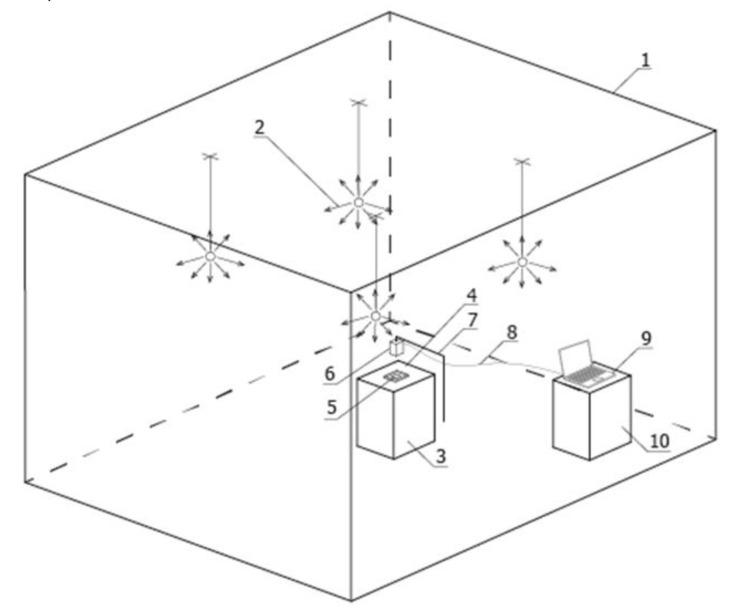
Schematic of test bench components.

Image registration and analysis of the luminance distribution on the surface of the test samples was carried out according to the following methodological approach:

The test bench for measuring surface luminance distribution was installed in a closed room, illuminated by electric light with precisely selected wall and ceiling reflectance (1) (Figure 1). 

**Figure 2:**
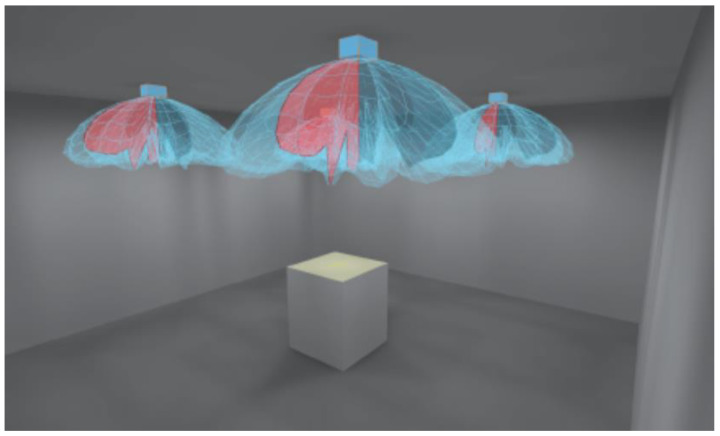
The luminary arrangement inside the room to ensure even illumination of the test bench.

A set of four luminaires (2), providing direct or indirect illumination to the test bench, was the light source. The measuring table (3) was coated with a black material (4) performing Lambertian reflection, absorbing the light flux falling on this material directly from the luminaires and indirectly from the ceiling and walls. Light sources (Figure 2) emitted a luminous flux with a colour temperature of 4000K and optics with a rotationally symmetric photometric solid and diffuse luminous flux distribution, creating direct or indirect illumination of the measuring plane on which the sample was located (5). This allowed the entire surface of the measuring table to be evenly illuminated. An evenness of luminance distribution of 98% was achieved.

The luminance distribution on the sample surface was measured using a small CMOS-based luminance matrix measuring head with a resolution of 4008 × 2672 pixels (6) placed on a tripod (7) centrally above the measuring table. The tripod provided a stable position and the precise positioning of the measuring head above the geometric centre of the measuring table surface. Another element of the test bench was the optical cable (8) connecting the luminance measuring device to the software on the computer (9) and the desk (10) on which the computer set was located.

The luminance distribution on the surface of the samples was measured on the rectangular plane of the measuring table (3). A base slide was placed on the black material (4), isolating the sample from the table measuring surface. A sample of the prostate gland tissue examined was placed on a slide. The test sample was pressed all over with a cover slide. 

**Figure 3:**
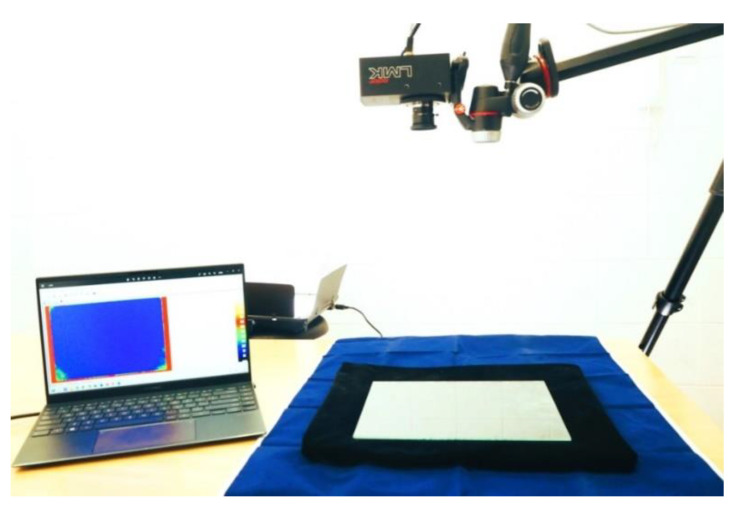
Photo showing test bench.

An actual picture of the test bench is presented in Figure 3.

The head of the luminance recorder was positioned directly above the sample, with the possibility of precisely correcting its position in three axes. Using an experimental method, the most favourable height of the digital recorder relative to the sample was determined, which was 58 cm. 

The height set was determined by the following method: covering the entire sample area with the registration field without excessive additional margins, obtaining the smallest possible sample area identified by a single pixel, limiting the measurement error associated with the registration angle of the image of the sample peripheral zone, and avoiding the phenomenon of uncontrolled shading of the sample with the recorder head.

Dedicated software for the measuring head was used to losslessly convert a photograph of the sample surface into a pseudo-colour image of the luminance distribution of the sample surface, allowing for further analysis to determine whether a given prostate tissue sample was free of neoplastic lesions or showed neoplastic lesions. 

LMK LabSoft software (version: 20.11.11) was used in the cognitive process [https://www.technoteam.de/products/software__add_ons/lmk_labsoft/index_eng.html, accessed on 14 December 2024].

**Figure 4:**
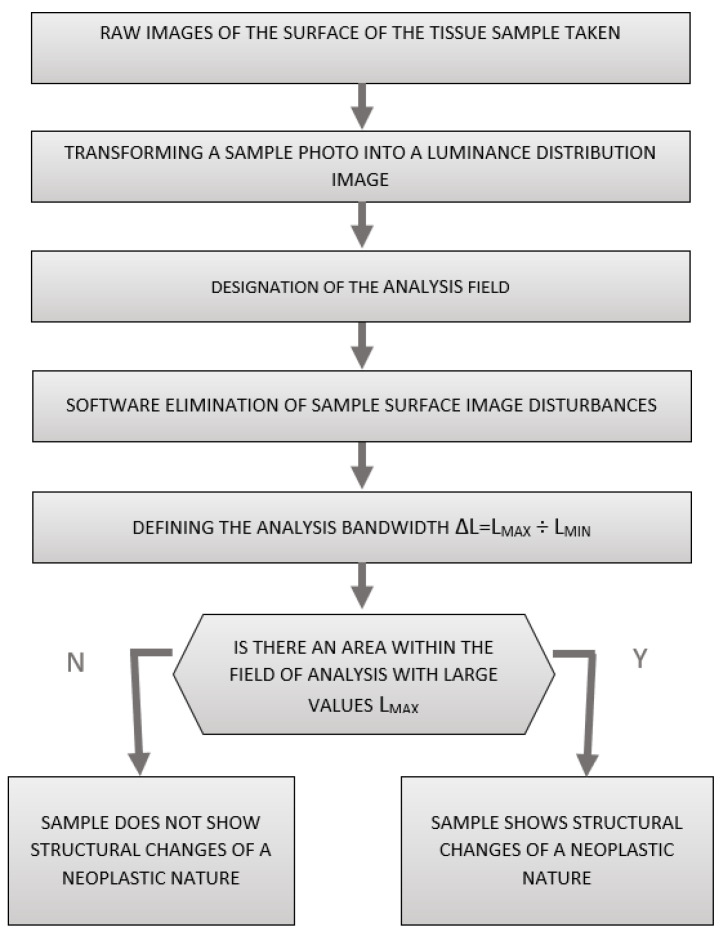
A flow chart of the process of the computer analysis of prostate gland samples using surface luminance decomposition.

A flow chart of the process of the computer analysis of prostate gland samples using surface luminance decomposition is shown in Figure 4.

The study of the luminance distribution of the sample surface involved the analysis of each pixel of the image taken by the recorder head. The test image was saved in the RAW format without any transformations that could introduce additional errors. In the next step, the luminance values of all image pixels were analysed and stored in the corresponding set.

**Figure 5:**
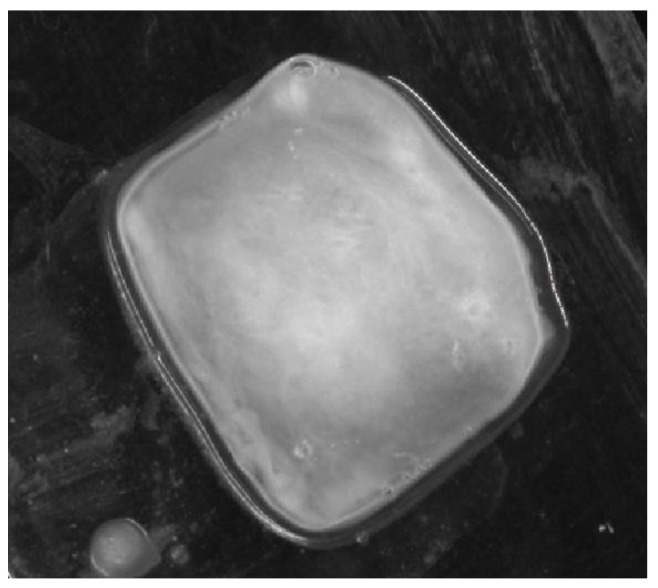
The monochromatic actual image of an example sample.

**Figure 6:**
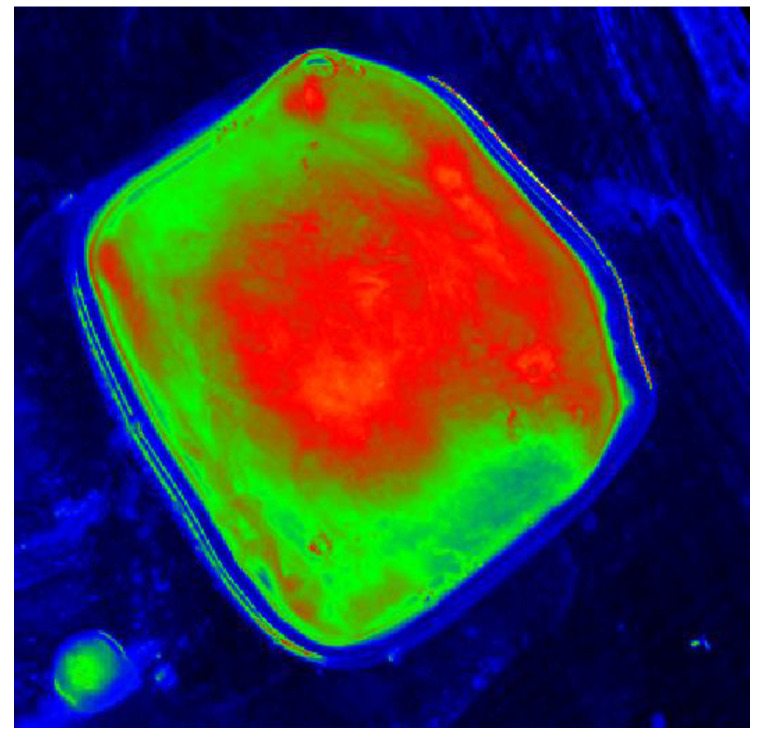
The software-generated high-resolution image of the luminance distribution of an example sample.

Next, the monochrome real image of the sample, Figure 5, was loaded into the LMK LabSoft software, which automatically generated a high-resolution image of the luminance distribution, Figure 6. The generated luminance image enabled the precise localisation of the foci, as well as the determination of their shape and surface area. High luminance values were characteristic of tissues with structures showing neoplastic lesions.

**Figure 7:**
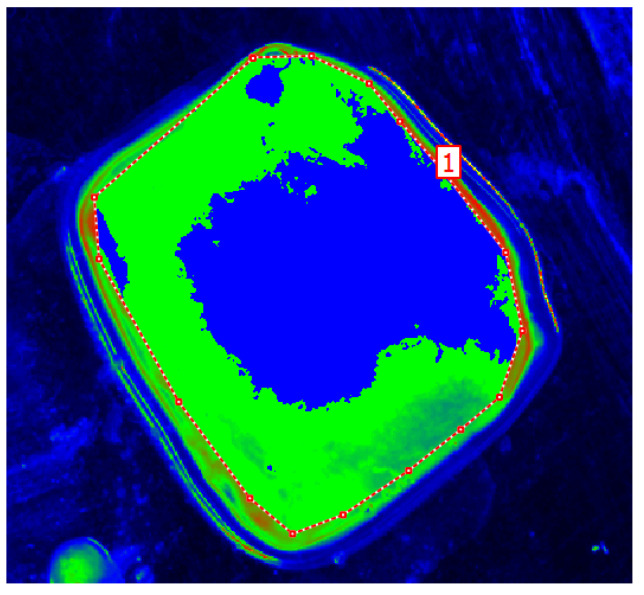
Pseudo-colour image generated with tissue surface without lesions (green) and with neoplastic lesions (blue).

The next step was to define the contour and the size of the measurement field and to set an appropriate cut-off threshold and generate the image in pseudo-colours. The key value defined during the study was the luminance value threshold, which allowed the separation of pixels via the colour range characteristic of tissue with no lesions (green) and tissue showing neoplastic lesions (blue), Figure 7. The corresponding sets of pixels were further counted. Once this was done, the software displayed a tabular result containing information on the mean, maximum and minimum luminance values.

The results obtained were statistically analysed to investigate whether cancerous lesions diagnosed within the prostate gland by measuring surface luminance distribution and by microscopic histopathological analysis as a reference method were related, and to determine the strength of the relationship. 

Forty-eight samples were used for statistical analysis. The verification tool sample size/area under ROC curve was used to determine the minimum sample size necessary to allow the statistical analysis of surface luminance distribution for prostate cancer assessment. Based on the calculations carried out according to an assumed error of inference I and II = 0.01 and a ratio of negative to positive results = 0.7, the minimum number of trials required to allow statistical calculations to be performed was 48 trials. 

In a statistical procedure to assess the diagnostic performance of the surface luminance distribution method in relation to the histopathological findings, an ROC curve was drawn. The area under the ROC curve was calculated, determining sensitivity and specificity rates with 95% confidence intervals. 

**Figure 8:**
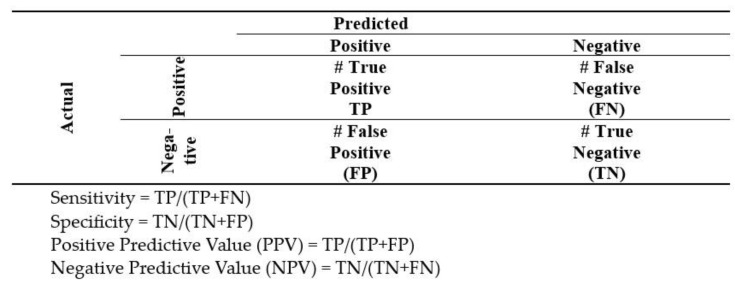
Confusion matrix and definitions for sensitivity, specificity, positive and negative predictive value.

To determine the diagnostic reliability of the luminance method in relation to the conventional histopathological reference method for the samples tested, the sensitivity and specificity values of the method used were assessed for different ranges of maximum luminance values. For the purpose of the analysis, the samples were divided according to the following scheme (Figure 8):

The detailed statistical processing included the determination of the following indicators: sensitivity, specificity, positive predictive value (PPV), negative predictive value, LR+, positive reliability quotient and LR- reliability.

Pearson’s non-parametric χ2 test was used to test the relationship between the two variables measured on the qualitative scale. A statistically significant result of *p* < 0.05 indicates the presence of a relationship between the analysed variables. Pearson’s χ^2^ coefficient was calculated with the formula

$$\chi^{2}=\sum_{i=1}^{k}\frac{(O_{i}-E_{i}{)}^{2}}{E_{i}}$$

where *O_i_*—observed count; *E_i_*—expected count. In addition, the strength of Cramer’s V compound was calculated as follows: $V=\sqrt{\frac{\chi^{2}}{n\cdot min(k-1,r-1)}}$, where *k* is the number of columns and *r* is the number of rows in the contingency table. 

The results of the statistical analysis carried out are summarised in tables containing the assignments of the surface luminance distribution of samples compared to the reference method and containing the values of the sensitivity, specificity and reliability indices of the test for different luminance thresholds. 

## 3. Results 

**Figure 9:**
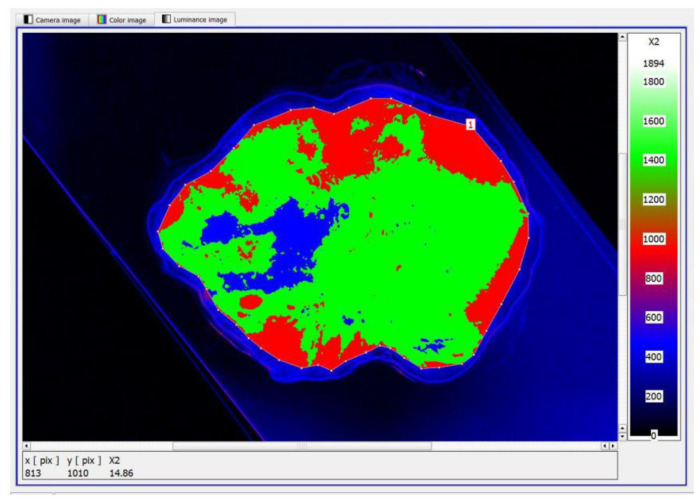
The analysis phase of the luminance distribution on the sample surface using dedicated computer software.

In the initial analysis of the results, the variants of structural lesions of the prostate gland tissue of a neoplastic nature identified during the study were presented, which differed in terms of area size, number of foci, location and their characteristic mean luminance values. Figure 9 shows an example of the luminance distribution on the surface of the prostate samples, generated by specialised software for luminance reading. The generated image will be used in a further step to search for cancer-lesion structures by using a software functionality that allows luminance bands to be defined. 

**Figure 10:**
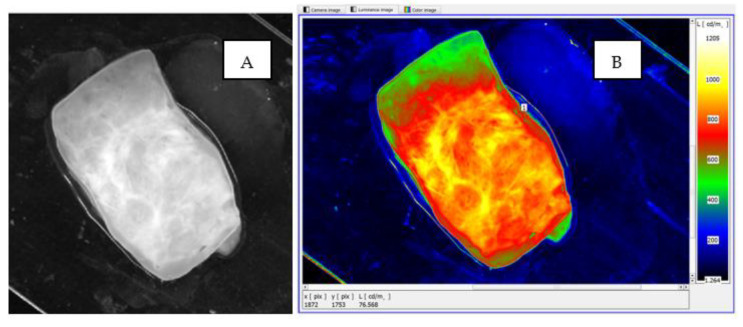
Images of sample surface. (**A**) Image of advanced neoplastic lesions, (**B**) image of advanced neoplastic lesions visible in pseudo-colour luminance distribution.

To assess the usefulness of imaging the sample surface in the form of a luminance distribution, a comparison was made between the actual image of the sample subjected to classical visual assessment by the morphologist and its processed luminance image, with a defined luminance distribution (Figure 10). The image of the sample surface shows an enlarged area of cancer cells, located in the central zone of the sample, which is marked with a dashed envelope. In both the real image (Figure 10A) and the luminance counterpart, there was convergence in the location, area size and shape of the neoplastic lesions. It is noteworthy that the luminance image, due to the use of pseudo-colour distribution, is much clearer and therefore easier to use to identify neoplastic lesions (Figure 10B).

In the illustrated case, the surface area of the cancer cells covers most of the specimen area. The mean luminance of the area with structural lesions is markedly elevated, accumulating closer to the central focus of disease (Figure 10B). The area affected by the structural lesions was found to have a significant increase in luminance to a level of approximately 900 cd/m^2^.

**Figure 11:**
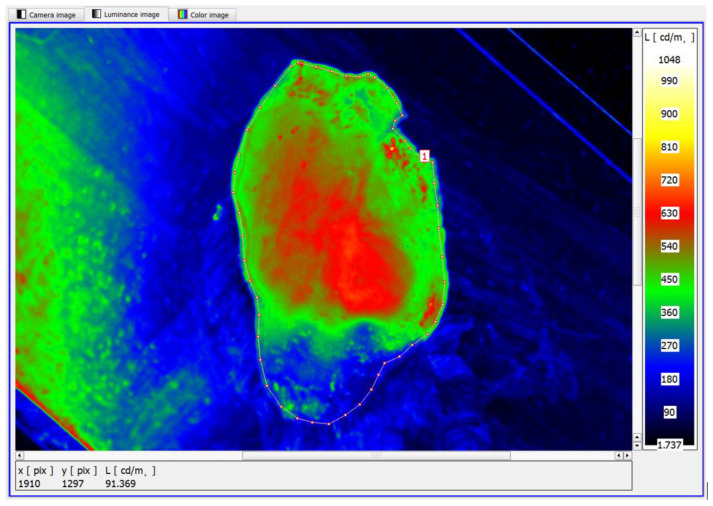
An example of a sample showing the developmental phase of a single focus—pseudo-colour image of the luminance distribution.

The study showed that the functionality of the software used to analyse the luminance distributions of the prostate specimens examined also offered the possibility of identifying smaller area foci of neoplastic lesions (Figure 11). 

**Figure 12:**
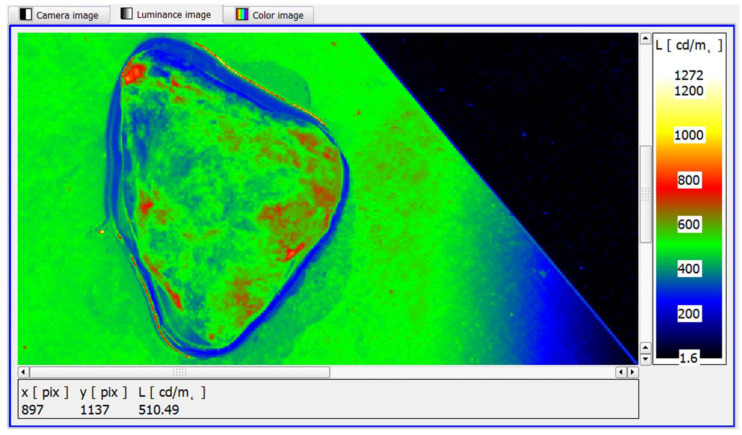
A sample with several peripherally localised cancer foci present in the peripheral strip of the specimen—pseudo-colour image of the luminance distribution.

In the example shown, the mean maximum luminance of the area with structural lesions was in the range of 630–660 cd/m^2^. Similarly, the luminance method has been shown to identify diffuse structural foci of neoplastic lesions (Figure 12). It is worth noting that the diffuse foci show approximate average luminances in the range of 680–720 cd/m^2^. 

This is a luminance level similar to the single-focal variant, where a single neoplastic lesion is present (Figure 11). 

**Figure 13:**
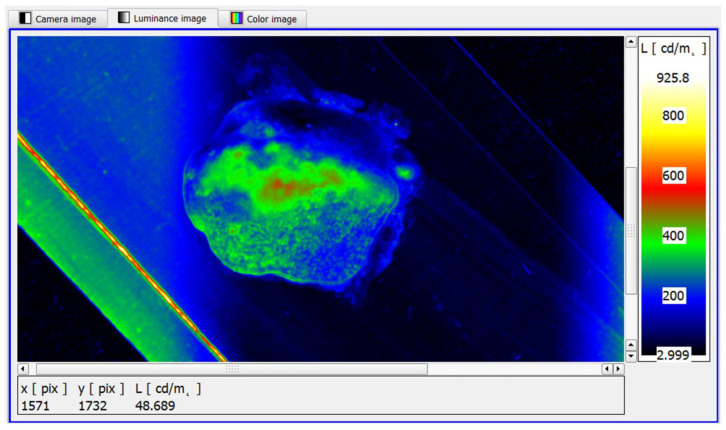
A sample case showing a small area located in the central zone of the sample with slightly increased luminance—a pseudo-colour image of the luminance distribution.

Further narrowing the range of the pseudo-colour distribution of luminance in the software makes it possible to take full advantage of its functionality, i.e., to identify small surface areas of the sample showing features of slightly increased luminance. In this case, through the clearer colour contrast produced, it is easier for the observer to assess the sample for the possible presence of cancer foci (Figure 13).

Analysing the test material, fragments with a small area were isolated, whose maximum luminance value was in the range of 500–520 cd/m^2^ (Figure 13). This demonstrated the absence of neoplastic lesions and allowed the test sample to be interpreted as healthy, which was confirmed by the reference method.

The analysis of the surface luminance distribution of the examined specimens shows that the structural changes in the tissue caused by the neoplastic agent are characterised by a different range of mean luminance, additionally conditioned by the surface area and the number of lesions, compared to the luminance distribution occurring on the surface of prostate samples without and with neoplastic lesions. The surface luminance of the lesioned samples ranged from 630 to 1130 cd/m^2^ (Figure 10, Figure 11, Figure 12). In contrast, the maximum luminance on the surface of the normal specimen in the histopathological evaluation was significantly lower, at around 500 cd/m^2^ (Figure 13).

This allowed statistical analysis to be carried out to establish a relationship between the mean luminances of the prostate gland specimens and the diagnosis made by the pathologist. 

Table 1 summarises the basic statistical indices characterising the maximum luminance that was recorded on the surface of the samples with and without neoplastic lesions. The maximum luminance for samples without lesions ranged from 1013 to 1206 [cd/m^2^], while that for samples with neoplastic lesions ranged from 1178 to 1202 [cd/m2], with arithmetic means of 1091 [cd/m^2^] and 1188.4 [cd/m^2^], respectively. It was found that the difference between the arithmetic means was statistically highly significant (*p* ≤ 0.0001) Table 1. It is noteworthy that a significantly higher range of standard deviation was recorded in the set of non-cancerous samples.

Further statistical analysis showed that the Glass biserial correlation coefficient was r = 0.61, which indicates that the relationship between the results obtained by the compared methods was strong.

The luminance values, Lmax [cd/m^2^], of the samples were used to assess the test result. The Lmax cut-off levels determining the assignment of samples without neoplastic lesions (negative result)/with neoplastic lesions (positive result) were selected based on the distribution of results for the following values: 1070 dc/m^2^, 1080 dc/m^2^, 1090 cd/m^2^, 1100 cd/m^2^, 1100 cd/m^2^, 1120 cd/m^2^ and 1130 cd/m^2^. The distribution of sample assignments for the cut-off points is shown in Table 2.

**Figure 14:**
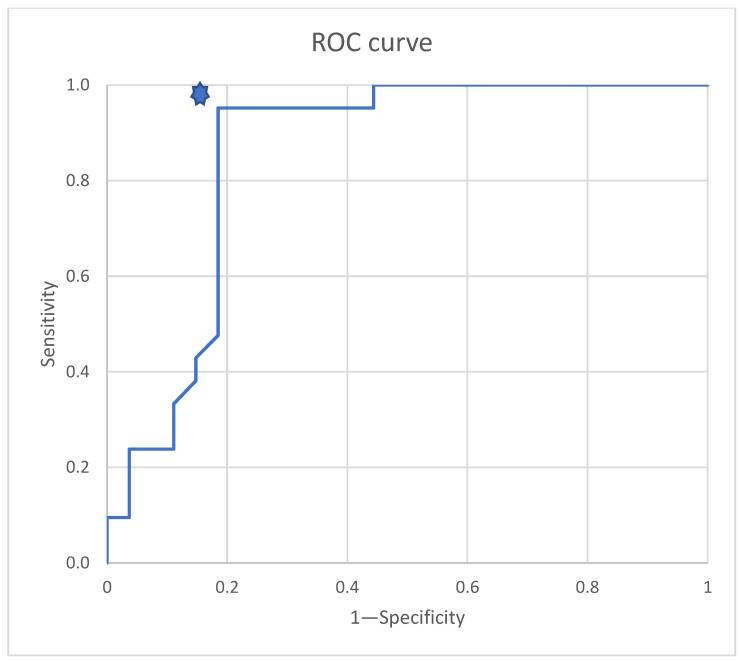
ROC curve for luminance measurements of samples.

In accordance with the analytical approach adopted, the relationship between sensitivity and the inverse of specificity was first illustrated for the Lmax values of the samples (Figure 14). Based on the analysis of the data, the best prediction was obtained for a test with a sensitivity of approximately 95%, a value of 1 and a specificity of approximately 18%, with a sample differentiation threshold of Lmax = 1093.5 cd/m^2^. Based on the data in Table 2, it was found that the best sensitivity and specificity values were obtained for the following thresholds: Lmax = 1090 cd/m^2^ and Lmax = 1100 cd/m^2^. These thresholds are the same due to the lack of samples in the range 1090 cd/m^2^ < Lmax < 1100 cd/m^2^. Finally, on the basis of the results obtained, the sample qualification threshold was adopted at a level of Lmax = 1100 cd/m^2^ (Table 2, Figure 14).

The highest values for sensitivity and specificity were at a luminance threshold of Lmax = 1100 cd/m^2^, and were 81.48% for sensitivity and 95.24% for specificity, respectively (Table 2). This means that when samples were split with a threshold of Lmax = 1100 cd/m^2^, 81.48% of positive samples and 95.24% of negative samples were correctly identified in relation to the histopathological reference method. Analysis by Pearson’s χ^2^ test confirmed that the result was statistically significant: χ^2^(1) = 27.86; *p* < 0.001; V = 0.76. It is noteworthy that when analysing higher or lower luminance thresholds, less accurate sensitivity and specificity indices were obtained (Table 2), which confirms the correct setting of the sample-matching threshold at Lmax = 1100 cd/m^2^.

From the data in Table 3, it can be seen that for a threshold of Lmax = 1100 cd/m^2^, the probability of a positive result obtained from a luminance measurement being consistent with a positive result obtained by histopathological examination was 95.65%. In contrast, the probability of concordance of the negative results of the compared methods was 80%. The calculated value $LR_{+}$ indicates that the chance of a positive luminance result in a sample with pathological tissue in relation to the chance of a positive luminance result for samples without cancerous lesions was, for a threshold of Lmax = 1100 cd/m^2^, 17.11.

It was further shown that the chance of a negative luminance result in a sample with neoplastic lesions relative to the chance of a negative luminance result in samples with negative results was 0.19. When the samples were split below and above the luminance threshold of Lmax = 1100 cd/m^2^, the reliability of the test was 87.50%. This means that such a luminosity threshold in the study group allowed the 87.50% correct separation of both negative and positive specimens in relation to the diagnosis by the reference histopathological examination method. 

## 4. Discussion

Matrix luminance meters have a wide spectrum of use [26]. In recent years, a number of studies have been initiated to use luminance measurement in the assessment of structural parameters of tissue material of plant and animal origin. According to Arce-Loper et al., measuring the exact luminance values of texture changes in selected vegetable species allows for the assessment of freshness and consumer appeal [29]. Other studies [27] have demonstrated the usefulness of luminance measurements for studying the tissue structure of meat and assessing marbling and intramuscular fat content. 

As is well known [12,15], numerous computer image processing and analysis techniques have been used in the diagnosis of neoplastic lesions for many years. The main task of computer image processing in diagnostic medicine is to help make disease-induced changes in the body as visible as possible and provide reliable diagnostic information [20,30]. 

The present study attempted to use luminance measurement to assess neoplastic lesions within the prostate gland, ranked among the most common and particularly aggressive male cancers [12,15]. It was assumed that differences between the normal and pathological anatomy and physiology of the prostate gland could, among other things, result in changes that would be visible in the images recorded by the matrix luminance meter. This assumption was positively verified during the course of the study.

The results obtained in our study indicate that the distribution of luminance on the surface of the prostate gland tissue samples in which neoplastic lesions were found according to histopathological findings is different compared to tissue without neoplastic lesions. The differences found are primarily due to the luminance value Lmax. The highest sensitivity and specificity values were obtained for a luminance threshold of Lmax = 1100 cd/m^2^. The reliability of the test at the designated threshold was 87.50%. It is worth noting the very high probability of a positive result obtained with the luminance measurement being as high as 95.65% compatible with a positive result obtained with the reference histopathological examination. 

The authors believe that a certain limitation at the present stage of the research is the unsatisfactory value of the method’s sensitivity coefficient, which is 81.48%, Table 2. Among the main possibilities that could contribute to improving the method’s sensitivity coefficient, improvement of the sample preparation procedure for measuring surface luminance distribution should be pointed out. The authors encountered the problem of removing moisture from the sample surface, which was accumulating under the cover glass, which generated light reflections, causing an error in the luminance reading. A similar problem was also pointed out by other authors [31]. An important factor contributing to the improved agreement between the results of the compared methods should be seen in the elimination of human error related to the precise positioning of the measuring head with respect to the sample surface. 

An overriding role in histopathological examinations is occupied by microscopic examinations, which involve the appropriate preparation of tissue specimens and their microscopic evaluation to determine the histological type of the tumour, the malignancy and the clinical progression of the condition. Despite the use of sophisticated equipment and modern reagents, the final interpretation of the results is up to the pathologist and depends on their skills and the amount of information available to them. 

The high efficiency, accuracy and measurement speed of the presented diagnostic method make it possible to consider the analysis of surface luminance distribution as a reliable and accurate tool supporting the correct diagnosis of neoplastic lesions within the prostate gland at the stage of histopathological examination. The essential added value of the proposed method over the classic histopathological examination is the introduction of an objective tool system in the diagnostic process, allowing the identification of lesions by analysing the luminance features of individual pixels. The measurement system can support the work of histopathologists by enhancing the accuracy and identification capabilities of differences inaccessible to the human eye. The method also remains insensitive to the fatigue factor and the risk of errors generated by the human factor. The authors note a particular opportunity to use the method for the histopathological evaluation of whole-mount histopathology (WMH) preparations. Recognised as the gold standard in PCa diagnosis, WHM analysis provides comprehensive histopathological information as well as spatial information, providing a reference method for molecular imaging, ultrasound and MMR imaging [32].

Hence, the authors point to the possibility of installing the measurement system as part of the equipment in histopathology laboratories. During the histopathological examination of preparations after radical prostatectomy, it is necessary to analyse multiple samples separated from a single gland removed by surgery. The findings should provide information on the following factors: current stage, local histopathological malignancy and the status of surgical margins of prostate cancer. Obtaining additional information from the analysis of the luminance distribution on the surface of the specimens can contribute to better identification of the topography of the neoplastic infiltration and can also speed up the histopathological evaluation. In addition, the compact size of the test bench allows for the solution to be recommended for use in intraoperative diagnosis in radical prostatectomy procedures. The proposed method can be an additional source of diagnostic information allowing the surgical oncologist to make the optimal decision regarding surgical intervention.

The direct relationship between surface luminance distribution and the histopathological and prognostic features of neoplastic lesions within the prostate gland demonstrated in our own study is an interesting relationship that may have practical diagnostic applications. At the same time, it should be emphasised that the research presented is preliminary. The authors are aware of the limitations of the method and the problems associated with its practical application. It should also be noted that this was a single-centre retrospective study that included only patients who had undergone radical prostatectomy for cancer. According to generally accepted guidelines applicable to the validation of new diagnostic methods, multi-centre studies are recommended [16]. A condition necessary for the validation of the presented method, aimed at eliminating potential errors, and ensuring that the collected information is reliable and credible is the continuation of the research aimed at collecting more data to allow in-depth statistical analysis. Preferably, the research should be conducted in different medical centres. In the case of compiling the results obtained from multi-centre clinical trials with similar baseline characteristics of the sites, there is a need for meta-analysis, which involves combining the results and conducting a full statistical analysis and obtaining more reliable results.

Therefore, the improvement of the presented diagnostic tool requires a multidirectional continuation of research. However, it should be emphasised that this is a natural state when developing new diagnostic techniques. Similarly, ref [33] points out that a number of intrinsic limitations of each of these techniques are identified during the testing and implementation phase, and further research is required before they are finally considered reliable tools for accurate PCa diagnosis. For example, other new technologies, such as elastography and multi-parametric ultrasound, are currently based on a limited number of verification studies; hence, the assessment of their practical usefulness requires continued research. A particularly interesting direction for further research should be to experimentally test whether surface luminance distribution can be used to identify neoplastic lesions in core samples taken during biopsy. A positive result of such studies would multiply the analytical potential of AI-enabled biopsy preparations [28]. Based on the literature [34,35], it can be concluded that AI-based software can improve the efficiency and accuracy of detecting clinically significant prostate cancer. Review papers [34,35] show that AI-based algorithms have improved the diagnostic performance of prostate cancer by interpreting information obtained by imaging diagnosis methods with TRUST ultrasound, MRI, MpMRI or based on the Gleason scale. Therefore, it can be assumed that there is a possibility of applying AI to analyse the results of prostate cancer lesions obtained by means of surface luminance distribution measurements. It is indicated that new technologies such as artificial intelligence (AI) and machine learning (ML) will be crucial in the field of prostate cancer diagnosis and treatment in the near future, including the analysis of histopathological samples [28]. 

A separate issue that can significantly increase the potential of the presented method is the evaluation of the possibility of applying surface luminance distribution in the diagnosis of cancers of other organs of the human body. It should be emphasised that the high accuracy and reliability of identifying neoplastic lesions on the surface of tissue sections of the prostate gland, proven by the study, can be a good predictor for the effectiveness of the histopathological evaluation of sections with suspected neoplastic lesions taken from other organs. However, this requires detailed clinical studies that take into account the specifics of individual cancers and their methods of diagnosis.

## 5. Conclusions

The tests carried out showed that the surface luminance distribution analysis method allows for the identification of neoplastic structural tissue changes in prostate samples. The method has a high reliability index of 87.50% and a 95.65% probability of compatibility with the results of the reference histopathological examination. The results indicate that the method can be a tool to help diagnose prostate cancer. The essential added value of the proposed method over the classic histopathological examination is the introduction of an objective tool system to assist histopathologists in the diagnostic process. The possibility of installing the measurement system in histopathology laboratories and as an additional tool in intraoperative diagnosis in radical prostatectomy procedures is indicated.

## Figures and Tables

**Table 1:** Statistical characteristics of maximum luminance [cd/m^2^] of samples with and without neoplastic lesions.

	N	Min.	Max.	M	SD	Q1	Me	Q3	Z	*p*	r
Without neoplastic lesions	21	1013	1206	1091.00	27.30	1116.5	1168.5	1192	4.17	0.0001	0.61
With neoplastic lesions	27	1178	1202	1188.40 *	19.02	1180.5	1189	1196			

* significant at *p* ≤ 0.05.

**Table 2:** The distribution of the assignment of luminance samples to the reference method.

			Reference Method	
			Positive (+)	Negative (−)
Lmax 1070 cd/m^2^	Positive result	N	18	1
		%	66.67%	4.76%
	Negative result	N	9	20
		%	33.33%	95.24%
Lmax 1080 cd/m^2^	Positive result	N	20	1
		%	74.07%	4.76%
	Negative result	N	7	20
		%	25.93%	95.24%
Lmax 1090 cd/m^2^	Positive result	N	22	1
		%	81.48%	4.76%
	Negative result	N	5	20
		%	18.52%	95.24%
Lmax 1100 cd/m^2^	Positive result	N	22	1
		%	81.48%	4.76%
	Negative result	N	5	20
		%	18.52%	95.24%
Lmax 1110 cd/m^2^	Positive result	N	22	4
		%	81.48%	19.05%
	Negative result	N	5	17
		%	18.52%	80.95%
Lmax 1120 cd/m^2^	Positive result	N	22	6
		%	81.48%	28.57%
	Negative result	N	5	15
		%	18.52%	71.43%
Lmax 1130 cd/m^2^	Positive result	N	22	7
		%	81.48%	33.33%
	Negative result	N	5	14
		%	18.52%	66.67%

**Table 3:** Sensitivity, specificity and reliability values of test for different luminance thresholds of samples.

	Sensitivity	Specificity	1—Sensitivity	1—Specificity	PPV	NPV	LR_+_	LR_−_	Credibility
Lmax 1070 cd/m^2^	66.67%	95.24%	33.33%	4.76%	94.74%	68.97%	14.00	0.35	79.17%
Lmax 1080 cd/m^2^	74.07%	95.24%	25.93%	4.76%	95.24%	74.07%	15.56	0.27	83.33%
Lmax 1090 cd/m^2^	81.48%	95.24%	18.52%	4.76%	95.65%	80.00%	17.11	0.19	87.50%
Lmax 1100 cd/m^2^	81.48%	95.24%	18.52%	4.76%	95.65%	80.00%	17.11	0.19	87.50%
Lmax 1110 cd/m^2^	81.48%	80.95%	18.52%	19.05%	84.62%	77.27%	4.28	0.23	81.25%
Lmax 1120 cd/m^2^	81.48%	71.43%	18.52%	28.57%	78.57%	75.00%	2.85	0.26	77.08%
Lmax 1130 cd/m^2^	81.48%	66.67%	18.52%	33.33%	75.86%	73.68%	2.44	0.28	75.00%

## Data Availability

Data available on request due to restrictions (privacy, ethical reasons).
